# The use of a multicellular *in vitro* model to investigate uptake and migration of bacterial extracellular vesicles derived from the human gut commensal *Bacteroides thetaiotaomicron*

**DOI:** 10.1002/jex2.93

**Published:** 2023-07-10

**Authors:** Amisha A. Modasia, Emily J. Jones, L. Marie‐Pascale Martel, Hélène Louvel, Pierre‐Olivier Couraud, L. Ashley Blackshaw, Simon R. Carding

**Affiliations:** ^1^ Quadram Institute Bioscience Rosalind Franklin Road Norwich Research Park Norwich UK; ^2^ Norwich Medical School Norwich Research Park University of East Anglia Norwich UK; ^3^ National Institute of Health and Medical Research (INSERM) 6 Place Tristan Bernard Paris France

**Keywords:** bacterial extracellular vesicles, brain, cell culture models, gut, trafficking

## Abstract

Bacterial extracellular vesicles (BEVs) are increasingly seen as key signalling mediators between the gut microbiota and the host. Recent studies have provided evidence of BEVs ability to transmigrate across cellular barriers to elicit responses in other tissues, such as the central nervous system (CNS). Here we use a combination of single‐, two‐ and three‐cell culture systems to demonstrate the transmigration of *Bacteroides thetaiotaomicron* derived BEVs (Bt‐BEVs) across gut epithelium and blood brain barrier (BBB) endothelium, and their subsequent acquisition and downstream effects in neuronal cells. Bt‐BEVs were shown to traffic to the CNS *in vivo* after intravenous administration to mice, and in multi‐cell *in vitro* culture systems to transmigrate across gut epithelial and BBB endothelial cell barriers, where they were acquired by both microglia and immature neuronal cells. No significant activation/inflammatory effects were induced in non‐differentiated neurons, in contrast to that observed in microglia cells, although this was notably less than that induced by lipopolysaccharide (LPS). Overall, our findings provide evidence for transmigration of Bt‐BEVs across gut‐epithelial and BBB endothelial cell barriers *in vivo* and *in vitro*, and their downstream responses in neural cells. This study sheds light onto how commensal bacteria‐derived BEV transport across the gut‐brain axis and can be exploited for the development of targeted drug delivery.

## Graphical abstract

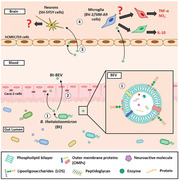

## 1 INTRODUCTION

Bacterial extracellular vesicles (BEVs) are nano‐size membranous vesicles released by both gram‐negative and gram‐positive bacteria which vary in origin, size, composition, and function (Kulp & Kuehn, 2010; Schwechheimer & Kuehn, 2015). Historically, their role has been associated with disease pathogenesis and the delivery of toxins and virulence factors produced by pathogenic parental bacteria (Bielaszewska et al., 2013; Bomberger et al., 2009). The role of BEVs produced by commensal bacteria is less clear, in particular those residing in the gastrointestinal tract (GIT) which hosts the largest population of bacteria in the body (Muraca et al., 2015; Stentz et al., 2018). Recent studies have demonstrated that BEV cargo includes an array of biomolecules including enzymes, metabolites and immunomodulatory proteins (Ellis & Kuehn, 2010; Fábrega et al., 2016; Vidakovics et al., 2010) suggesting they can influence various host cell functions (Bielaszewska et al., 2013). These properties together with the inherent stability of BEVs and their resistance to physical and (bio)chemical insult has led to their use as biologics and vaccine antigen delivery vehicles (Carvalho et al., 2019; Wang et al., 2019).

The presence of of BEVs in the blood (Jang et al., 2015; Jones et al., 2021; Park et al., 2017) indicates their ability to target and access tissues beyond the GIT. We confirmed this in our recent work (Jones et al., 2020) demonstrating the *in vivo* biodistribution of BEVs and accumulation in the liver and lungs following oral administration (Jones et al., 2020). Recent studies have also suggested the potential role of BEVs as long distance mediators of the gut‐(microbome)‐brain axis (GBA) (Kulp & Kuehn, 2010; Schwechheimer & Kuehn, 2015), providing evidence of interactions with peripheral pathways involved in gut‐brain communication (Al‐Nedawi et al., 2015; Lee et al., 2020). A direct influence on the central nervous system (CNS) (Ha et al., 2020; Han et al., 2019) is suggested by the finding that oral administration of BEVs from *Lactobacillus rhamnosus* (*L. rhamnosus*) strain JB‐1 can replicate the psychoactive effects of the parent bacterium (Al‐Nedawi et al., 2015; Bravo et al., 2011; Perez‐Burgos et al., 2013).

An important barrier to entry into the CNS from the circulation is the blood brain barrier (BBB) separating the cerebral capillary blood from the interstitial fluid of the brain and comprising endothelial cells, astrocytes, and pericytes embedded in the capillary basement membrane (Ballabh et al., 2004). *Haemophilus influenza* (*H. influenza*) type B‐derived BEVs have been shown to increase BBB permeability in rats (Wispelwey et al., 1989). BEVs derived from the periodontal pathogen *Aggregatibacter actinomycetemcomitans* were shown to traffic to the brain following intracardiac or intravenous administration where they localised to brain microglia cells, activating the proinflammatory nuclear factor kappa beta (NFκB) signal transduction pathways and production of the pro‐inflammatory cytokines tumour necrosis factor‐α (TNF‐α) and interleukin (IL)‐6 (Ha et al., 2020; Han et al., 2019). Consistent with a potential pathogenic role of BEVs is the finding that BEVs isolated from faecal microbiota of Alzheimer's Disease patients administered to mice daily for 8‐weeks resulted in disruption of the BBB, accompanied by increased activation of microglia and astrocytes, secretion of TNF‐α and IL‐1β together with tau phosphorylation resulting in cognitive impairment (Wei et al., 2020).

To address the biodistribution of BEVs derived from commensal gut bacteria and their ability to access the CNS we first sought evidence of their ability to access the CNS *in vivo* and second, used an *in vitro* multi‐cell culture system to investigate their trafficking between, and uptake by, intestinal epithelial cells, endothelial cells of the BBB and CNS‐derived microglia and neuronal cells. In contrast to numerous studies investigating BEVs produced by pathogens we have undertaken a study of BEVs produced by a commensal *Bacteroides spp*. that is a prominent member of the human intestinal microbiota.

## 2 MATERIALS AND METHODS

### 2.1 Bacterial cultures and BEV isolation

BEV preparation from *Bacteroides thetaiotaomicron* (Bt) VPI‐5482 (DSM 2079) and *Bacteroides fragilis* (Bf, DSM2151) cultures was modified from that previously described (Jones et al., 2020). The cells were cultured from frozen stocks in 10 mL of BDMr media containing 2.61 g KH_2_PO_4_ (Sigma, P5655), 7.03 g K_2_HPO_4_•3H_2_0 (Sigma, C7902), 15 mM NaCl (Sigma, 31434), 8.5 mM (NH_4_)_2_SO_4_ (Sigma, 31119), 30 mM glucose (Sigma, G7021), 0.1 mM MgCl_2•_6H_2_O (Sigma, M2393), 50 μM CaCl_2_•2H_2_O (Sigma, C7902), 0.2 mM L‐Histidine (Sigma, H8125), 2 μM Hemin (Sigma, 51280), 100 nM vitamin B12 (Sigma, V2876), 6 μM vitamin K3 Menadione (Sigma, M5625), 4.1 mM L‐cysteine hydrochloride (Sigma, W778567) and 1.4 μM FeSO_4•_7H_2_O in deionised H_2_O under anaerobic conditions at 37°C. Cells were cultured for 24 h to an optical density at 600 nm (OD_600_) of 1–2. The cultures were then used to innoculate 200 or 500 mL of the same media at 1:100 dilution and grown for 20–24 h to an OD_600_ or 1–2. BEVs were harvested from bacterial cultures by centrifugation at 6037 × g for 50 min at 4°C. Supernatants were then vacuum filtered using a 0.22 μM pore‐size polyethersulfone membrane (Sartorius, 180C5) to remove any contaminating cells and cellular debris. The supernatants were then concentrated to 5 mL by crossflow ultrafiltration (100 kDa MWCO, Vivaflow 50R, Sartorius) and the retentate rinsed once with 500 mL PBS (pH 7.4). The BEV suspensions were subsequently centrifuged at 15000 × g for 20 min at 4°C to remove any precipitate. The vesicles were then sterilised and filtered using a 0.22 μM syringe filter and quantified using nanoparticle tracking analysis (ZetaView, Particle Matrix).

### 2.2 Nanoparticle tracking analysis

ZetaView Nanoparticle tracking analyser (ParticleMetrix) with ZetaView (version 8.05.12 SP1) software running a 2 cycle 11 position high frame rate analysis at 25°C was used to quantify Bt‐BEVs and Bf‐BEVs. Sample dilutions were prepared in 1 mL ultra‐pure H_2_O prior to analysis to fit within an optimal detection range. Camera control settings used were: 80 Sensitivity; 30 Frame Rate; 100 Shutter. Post‐acquisition parameters: 20 Min Brightness; 2000 Max Area; 5 Min Area; 30 Tracelength; 5 nm/Class; 64 Classes/Decade. For detection of fluorescent‐labelled BEVs (488 nm), samples were diluted in 1 mL ultra‐pure H_2_O. With the fluorescent filter mounted, 1 mL of diluted sample was injected. The following measurements were used: run video acquisition > multiple acquisitions, low bleaching, dose sub volume, number of experiments = 11, number of cycles = 1. Analysis of 488 nm particles was recorded.

### 2.3 BEV labelling

For lypophyilic dye labeling, Bt‐BEVs (1 × 10^12^/mL) were incubated with 5% (v/v) 1,1′‐dioctadecyl‐3,3,3′,3′‐tetramethylindocarbocyanine perchlorate (DiD) and 3,3‐dioctadecyloxacarbocyanine perchlorate (DiO) (Molecular Probes, V‐22886, V22887) at 37°C for 30 min. Unbound dye was removed by washing three times with PBS using Amicon Ultra 0.5 mL centrifugal filters (100 kDa, Merck). For labelling BEVs with the protein dye Alexa Fluor (AF488/AF647), the total protein content of Bt‐BEVs was first determined using BCA protein assay (Abcam, ab207002) and adjusted to a final concentration of 2 mg/mL (Figure S1). Bt‐BEVs were diluted in 0.1 M sodium bicarbonate and incubated with AlexaFluor™ 488/647 reactive dye (ThermoFisher, A20173, A10235) with gentle stirring for 1 h at 22°C followed by size exclusion chromatography (Bio‐Rab BioGel P‐30 fine) to remove free‐dye. The size and distribution of labelled Bt‐BEV was determined by nanoparticle tracking analysis (ZetaView).

### 2.4 Animal experiments

Male and female specific pathogen‐free (C57BL/6‐SPF) mice were maintained in individually ventilated cages (SPF) in the University of East Anglia Disease Modelling Unit. All mice received autoclaved water and were fed RM3 (SPF) or RM3‐(Autoclavable) (GF) diet (Special Diets Services). Animal experiments were conducted in full accordance with the Animal Scientific Procedures Act 1986 under UK Home Office (HO) approval and HO project license 70/8232.

### 2.5 *In vivo* biodistribution imaging

**FIGURE 1:**
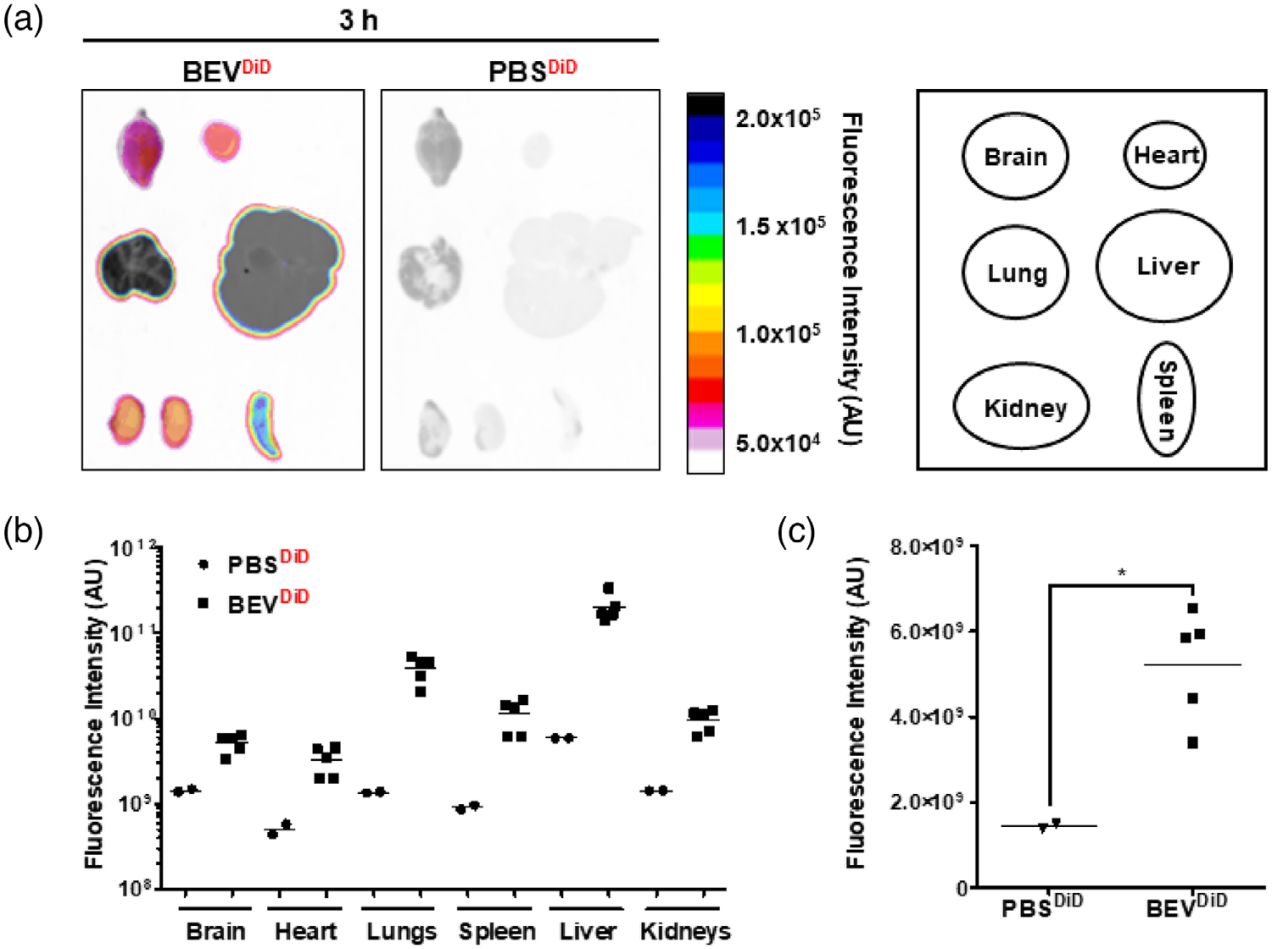
Biodistribution of fluorescent (DiD)‐labelled Bt‐BEVs in vivo. Adult SPF mice were administered intravenously with 2 × 1010 DiD‐labelled Bt‐BEVs (*n* = 5) or DiD‐PBS (*n* = 2). Tissues were excised 3 h later prior to imaging using a Bruker in‐vivo Xtreme imaging system (a). The graph in (b) depicts the sum of fluorescence signal detected in each organ. The graph in (c) depicts the fluorescence signal obtained from intact brain samples. Statistical significance was calculated using Student's unpaired *t*‐test (GraphPad Prism 5.04), with a value of *p* < 0.05 considered statistically significant; *p < 0.05.

Two‐month‐old female C57BL/6 mice were intravenously administered DiD‐labelled BtBEVs at a dose of 2 × 10^10^ BEV/mouse (*n* = 5) or PBS (*n* = 2) in a total volume of 200 μL/mouse. Organs including brain, heart, lungs, spleen, liver, and kidneys were excised at 3 h post administration and far‐red fluorescence acquired using an *in vivo* Xtreme multi‐modal optical and x‐ray small animal imaging system (Bruker) equipped with a back‐illuminated 4MP CCD detector. Foreground far‐red DiD fluorescence was recorded with the following settings: excitation 650 nm and emission 700 nm, 19 cm field of view, 20 s exposure time, fStop 1.1, and focal plane 0. Background image was recorded by reflectance as above with an exposure time of 1 s. Radiant efficiency of each organ was recorded using Bruker Molecular Imaging software (v 7.2.0.21148). Image analysis was performed using Image J/FIJI v1.52p by overlaying foreground and background images and recording organs as individual regions of interest (ROI, Figure 1). Data were displayed as mean arbitrary fluorescence units of each group.

### 2.6 Mammalian cell lines

The human cerebromicrovascular endothelial cell line, hCMEC/D3 (INSERM) (Weksler et al., 2005) was cultured at 37°C in 5% CO_2_ in endothelial cell growth basal medium‐2 (EBM‐2, Lonza) supplemented with EBM‐2 endothelial SingleQuots (Lonza) and 1% penicillin/streptomycin (Sigma) at passage 21–30. For co‐culture experiments cells were cultured in media without vascular endothelial growth factor (VEGF) to enhance expression of cell junction proteins (Cristante et al., 2013). The human colonic epithelial cell line, Caco‐2 (ECACC‐86010202), was cultured at 37°C in 5% CO_2_ in MEM (Sigma) supplemented with 10% fetal bovine serum (FBS) (Sigma), 1% L‐glutamine (Sigma), 1% penicillin/streptomycin at passage 19–25. BV‐2 microglia (AMSBIO) cells were cultured at 37°C in 5% CO_2_ in MEM (Sigma) supplemented with 10% FBS (Sigma) and 1% penicillin/streptomycin (Sigma) at passage 4–12. SIM‐A9 microglia (Kerafast Inc.) cells were cultured at 37°C in 5% CO_2_ in DMEM:F12 (ThermoFisher) supplemented with 1% penicillin/streptomycin, 10% FBS (Sigma), and 5% horse serum (Sigma) at passage 4–10. Non‐differentiated neuronal cells SH‐SY5Y (ECACC‐94030304) cells were cultured in EMEM supplemented with 1% L‐glutamine (Sigma), 1% penicillin/streptomycin, and 15% FBS (Sigma) at passage 16–22.

#### 2.6.1 Single‐and multi‐cell cultures

**FIGURE 2:**
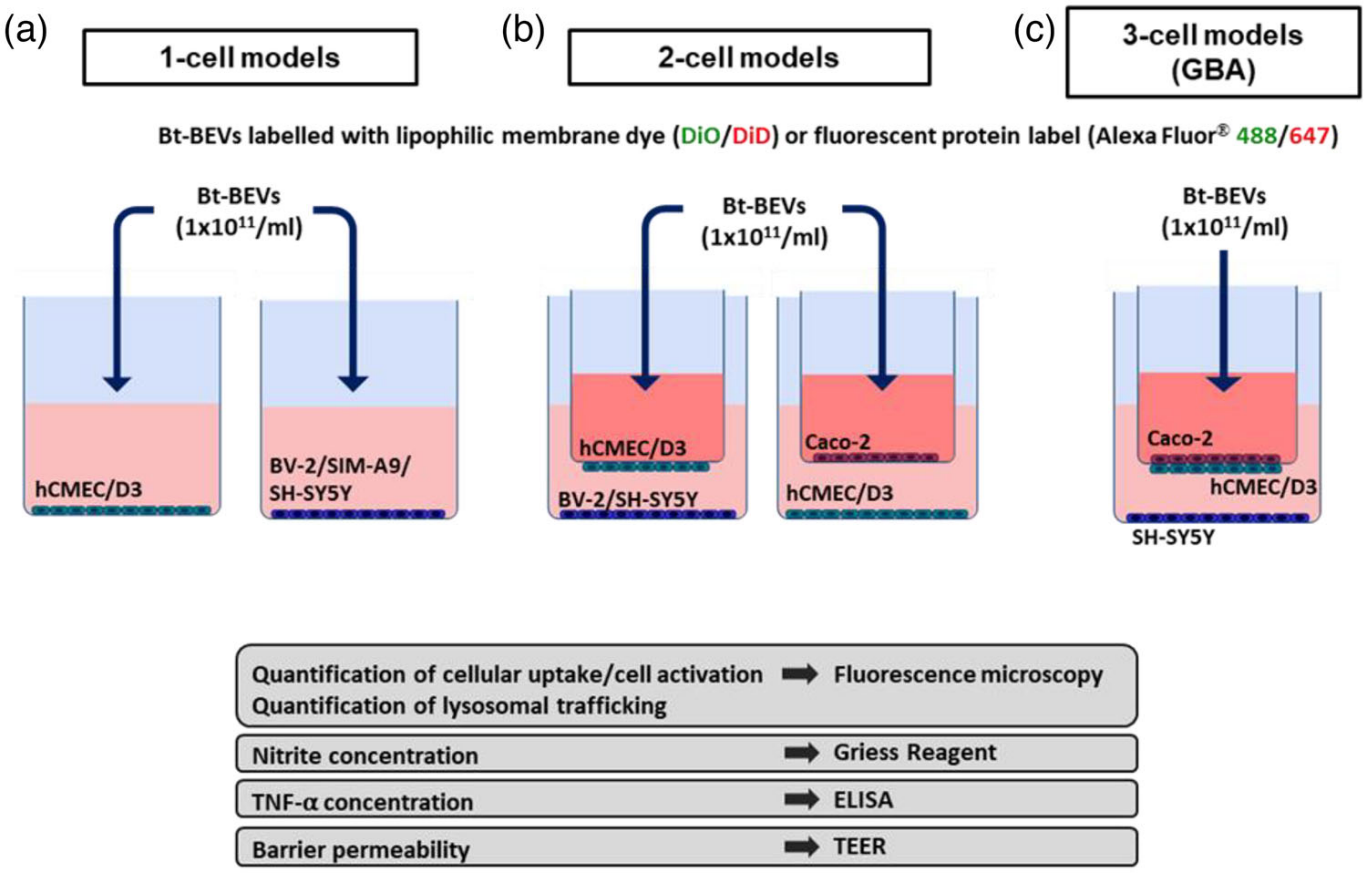
Experimental design of one‐, two‐ and three‐cell culture systems to assess uptake and transport of Bt‐BEVs across the gut‐brain axis. (a) One‐cell cultures modelling Bt‐BEV trafficking from the blood. hCMEC/D3 cells used to model the BBB endothelium, BV‐2/SIM‐9 cells used to model microglia and non‐differentiated SH‐SY5Y cells used to model immature neurons (b) For two‐cell cultures modelling the gut‐blood‐BBB, Caco‐2 cells were cultured on the apical surface of ThinCert inserts for 21 days to promote polarity and differentiation. hCMEC/D3 cells were cultured on collagen‐coated glass coverslips for 4 days and subsequently placed in the corresponding basal compartment of 24‐well culture plates. For the two‐cell BBB‐CNS model, hCMEC/D3 cells were allowed to adhere for approximately 16 h to the basal surface of ThinCert PET membranes which were non‐coated or coated with Matrigel. Subsequently, the ThinCert insert was placed in cell free media and cultured for a further 4 days. BV‐2 cells and SH‐SY5Y were cultured on either non‐coated or fibronectin coated glass coverslips in the corresponding basal compartment of 24‐well culture plates. (c) For the three‐cell culture to model transport across the gut‐blood‐brain axis, Caco‐2 cells were cultured on the apical surface of ThinCert membrane for 21 days. hCMEC/D3 cells were allowed to adhere for ∼16 h on the basal surface of Matrigel coated or non‐coated PET membranes before being placed in cell‐free media for a further 4 days. SH‐SY5Y cells were cultured for 24 h on fibronectin‐coated glass coverslips before being placed in the corresponding basal compartment of the 24‐well culture plate. Bt‐BEVs (1 × 10^11^/mL) labelled with lipophilic dyes (DiO/DiD) or Alexa FluorTM fluorescent protein (AF488/647) were added to cell culture systems for 24 h (37°C, 5% CO_2_). To assess cellular responses to Bt‐BEVs, fluorescent microscopy, Griess Reagent, ELISA and TEER measurements were conducted to quantify cellular uptake/activation, oxidative stress, inflammatory cytokine secretion (TNF‐ α/IL‐10) and barrier permeability, respectively.

The experimental design of the one‐, two‐ and three‐cell culture systems are depicted in Figure 2. For cell monocultures hCMEC/D3 (3 × 10^4^ cells/mL cultured for up to 4 days), BV‐2/SIM‐A9 (1 × 10^6^ cells/mL cultured for 24 h) and SH‐SY5Y cells (5 × 10^4^ cells/mL cultured for 24 h) were cultured on 1:20 collagen‐coated (3 mg/mL, Thermo Fisher), non‐coated or fibronectin (1 mg/mL, Thermo Fisher) coated 12‐well chamber slides (IBIDI) or glass coverslips in 24‐well culture plates (Figure 2a). For two‐cell cultures modelling the gut‐blood‐BBB, Caco‐2 cells (1 × 10^6^ cells/mL) were cultured on the apical surface of 0.4 μM PET membranes of ThinCert inserts (Greiner Bio‐one) for up to 21 days to promote polarity and differentiation. hCMEC/D3 cells (3 × 10^4^ cells/mL) were cultured on 1:20 collagen‐coated glass coverslips for up to 4 days and subsequently placed in the corresponding basal compartment of 24‐well culture plates. For the two‐cell BBB‐CNS model, hCMEC/D3 cells (3 × 10^4^ cells/mL) were allowed to adhere for approximately 16 h to the basal surface of 0.4 μM ThinCert PET membranes which were non‐coated or coated with 1:20 Corning Matrigel Growth Factor Reduced (GFR) Basement Membrane Matrix (Life Sciences). Subsequently, the ThinCert PET membrane was placed in cell free media and cultured for a further 4 days. BV‐2 cells and SH‐SY5Y were cultured on either non‐coated or fibronectin (1 mg/mL) coated glass coverslips in the corresponding basal compartment of 24‐well culture plates. For the three‐cell culture model, Caco‐2 cells (1 × 10^6^ cells/mL) were cultured on the apical surface of 0.4 μM PET membranes of ThinCert inserts for ∼21 days. hC MEC/D3 cells (3 × 10^4^ cells/mL) were allowed to adhere for approximately 16 h on the basal surface of Matrigel coated or non‐coated PET membranes before being placed in cell‐free media for a further 4 days. SH‐SY5Y cells were cultured for 24 h on fibronectin‐coated (1 mg/mL) glass coverslips before being placed in the corresponding basal compartment of the 24‐well culture plate.

### 2.7 Antibody staining and fluorescence microscopy

Fluorescently labeled Bt‐BEVs (at 1:10 dilution to yield a final concentration of 1 × 10^11^/mL), PBS or *Escherichia coli* derived LPS (25, 50, and 100 ng/mL) were added to the apical media of one‐ two‐ or three‐cell cultures for 24 h at 37°C in 5% CO_2_ (final volume 1 mL in well of one‐cell cultures and 250 μL in apical compartment of ThinCert inserts). Cells were fixed with PBS/4% paraformaldehyde, permeabilised with 0.25% Triton‐X100 in PBS (Sigma) and incubated with 10% goat serum (Sigma, G9023). Cultures were then incubated with Alexa 488‐phalloidin (1:1000, Thermo Fisher) to visualise intracellular membranes, anti‐LAMP1 (1:100, Santa Cruz Biotechnology) antiodies for lysosomes, recombinant rabbit monoclonal anti‐Iba‐1 (1:500, Abcam) for microglia activation, recombinant rabbit monoclonal anti‐TREM119 antibody (1:250, Abcam) and recombinant rabbit monoclonal anti‐CD45 antibody (1:250, Abcam) for microglia markers, rabbit polyclonal anti‐PCNA (1:500, Abcam), anti‐NeuroD1 (1:250, Abcam) and goat anti‐rabbit IgG (1:250, Abcam). Cells were then washed with PBS and incubated with Alexa Fluor594 goat anti‐rabbit Ig (1:1000, Invitrogen) or Alexa Fluor488 goat anti‐rabbit IgG (1:1000, Abcam). Hoechst 33342 nuclear stain (1:2000, Thermo Fisher) was used for nuclear visualisation and cells were mounted using Invitrogen™ ProLong™ Diamond Antifade mountant (Thermo Fisher). Cells were imaged using a Zeiss Axio Imager M2 microscope equipped with 40x/air objective and Zen blue software (Zeiss). In addition, Zeiss LSM880 confocal microscope equipped with 63x/1.4 oil DIC objective and Zen black software (Zeiss) was used to obtain higher resolution images. Fluorescence was recorded at 405 nm (blue), 488 nm (green), 594 nm (red) and 647 nm (far‐red) and intensity quantified using sum fluorescent pixel intensity of the field of view (FOV) normalised to nuclear counts using a macro written in Image J/FIJI v1.52p. Quantification of cell numbers were performed manually on ImageJ/FIJI v1.52p software using raw, unprocessed images. A minimum of 20 FOV images were used for any subsquent quantification. Co‐localisation was quantified by calculating the percentage of interalised BEVs that were co‐localised (using images representing different fluorescent channels, with yellow in merge channels representing co‐localisation) using a total of 20 fields of view from five biological replicates.

### 2.8 Transepithelial/transendothelial resistance (TEER)

Caco‐2 monolayers were seeded onto the apical compartment of the ThinCert PET membrane until fully confluent and TEER measurements recorded using an EVOM2 epithelial voltmeter with chopstick electrode (World Precision Instruments Inc.). Bt‐BEVs (at 1:10 dilution to yield a final concentration of 1 × 10^11^/mL) or PBS were added to the apical compartment (final volume 250 μL) and TEER measurements obtained at intervals of 3–16 h over a 24 h period. To optimise culture conditions to obtain highest TEER mesaurements, hCMEC/D3 cells were seeded on either collagen‐coated, Matrigel‐coated or non‐coated membranes of ThinCert inserts and TEER measurements recorded daily for 9 days.

### 2.9 Nitrite production

Nitrite (NO_2_^−^) concentrations were determined using the Griess Reagent (Promega). A nitrite standard reference curve was generated for each assay using 0.1 M soldium nitrite. Briefly, conditioned media samples were centrifuged to remove cell debris, aliquoted and stored at −20°C prior to use. Duplicate 50 μL of freshly thawed samples were added to wells of 96‐well flat‐bottom enzymatic assay plates followed by 50 μL sulphanilamide solution (1% sulfanilamide in 5% phosphoric acid) and incubated in the dark at 22°C for 5–10 min. Subsequently, 50 μL N‐1‐naphthylethlenediamine dihydrochloride (NED; 0.1% NED in water) was then added and incubated in the dark at 22°C for 5–10 min. Absorbance velues of 520–550 nm were recorded within 30 min using a microplate spectrophotometer (Bio‐Rad Benchmark Plus).

### 2.10 TNF‐α and IL‐10 production

TNF‐α and IL‐10 in cell conditioned media was measured using Invitrogen™ TNF‐α mouse ELISA (Thermo Fisher, 88‐7324‐88) and Invitrogen™ IL‐10 mouse ELISA (ThermoFisher, 88‐7105‐88) according to manufacturer's protocols.

### 2.11 Statistical analysis

All data are presented as mean ± standard error of the mean (SEM) with the indicated sample sizes. Box plots represent first quartile, median and third quartile, with whiskers representing minimum and maximum. For TEER, fluorescent intensity, nitrite, TNF‐α and cell count data, *p*‐values were calculated using either Mann–Whitney or Kruskal–Wallis tests. For IL‐10 datasets *p*‐values were calculated using Bonferroni's multiple comparisons test. For the *in vivo* Bruker biodistribution data, *p*‐values were calculated using Student's unpaired *t*‐test. All statistical analysis was carried out in GraphPad Prism 5 software (version 5.04), with a value of *p* < 0.05 considered statistically significant.

## 3 RESULTS

### 3.1 Bt‐BEVs localise to the mouse brain following intravenous administration

Evidence of Bt BEVs trafficking to the CNS was first sought from *in vivo* experiments in which DiD‐labelled Bt‐BEVs (2 × 10^10^/mouse) were administered intravenously to 2‐month‐old SPF‐mice. A control group received DiD‐PBS. Imaging of excised tissues 2 h later identified a fluorescent signal in the lungs, liver, kidneys, spleen, heart, and brain with the strongest signal detected in the liver (Figure 1b). The signal detected in the brain of BEV administered animals was weak compared to other tissues but significant when compared to vehicle (PBS) treated animals (Figure 1c). It was not possible to detect a signal in any tissues of PBS treated animals.

### 3.2 *In vitro* models of the gut‐BBB‐brain axis

*In vitro* cell culture models utilising one‐, two‐, and three‐cell culture systems were used to assess the trafficking to, acquisition by and impact of Bt‐BEVs on major epithelial and endothelial barrier cells and neuronal cells that are part of the gut‐brain axis (Figure 2). A cell monoculture system was used to assess uptake, intracellular fate, and immunomodulatory responses of individual cell types to Bt‐BEVs using BBB endothelial cells (hCMEC/D3), microglia cells (SIM‐A9 and BV‐2) and non‐differentiated neurons (SH‐SY5Y) (Figure 2a). *B. fragilis* BEVs were used as a comparator for Bt‐BEVs in the assessment of microglia responses. Cellular uptake, oxidative stress, inflammatory responses and barrier permeability were assessed using fluorescence microscopy, Griess reagent, ELISA and TEER, respectively.

### 3.3 Bt‐BEVs cross the gut epithelial barrier and enter BBB endothelial cells

We have previously shown uptake of BEVs by Caco‐2 cells (Jones et al., 2020). To determine if BEVs can also be acquired by BBB cells, Bt‐BEVs labelled with either the lipophilic dye DiD‐ or the protein dye AF674 were incubated with the BBB endothelial cell line hCMEC/D3 in a cell monoculture system (Figure 2) and visualised by confocal microscopy. BEVs were observed in the cytoplasm accumulating in the periplasmic space surrounding the nucleus of hCMEC/D3 cells (Figure S2A and B). BEVs were detected in various focal planes of the imaged cells consistent with their intracellular distribution (Figure S3). No 647 nm fluorescence signal was observed in PBS‐treated cells (Figure S2C). The proportion of Bt‐BEV^+^ cells per FOV was significantly higher (*p* < 0.01) when incubated with AF647‐labelled Bt‐BEVs compared to DiD‐labelled Bt‐BEVs (36.24 ± 2.08% and 21.37 ± 1.41%, respectively). Counterstaining with anti‐LAMP1 antibodies revealed that a significant proportion of internalised Bt‐BEVs (36.70 ± 3.47%) co‐localised with cellular lysosomes (Figure S2E), consistent with sequestration of BEVs by the endo‐lysosomal pathway.

**FIGURE 3:**
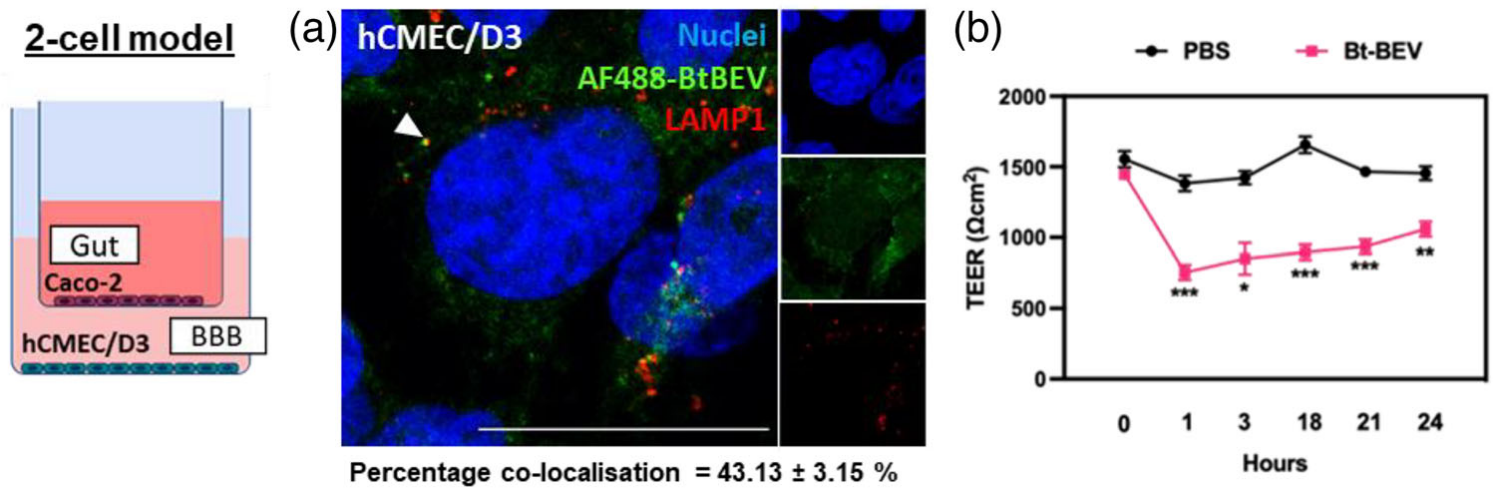
Bt‐BEVs cross the gut‐epithelial barrier and are acquired by BBB endothelial cells. Caco‐2 cells were cultured on ThinCert inserts (PET membrane, 0.4 μm) and hCMEC/D3 cells cultured on collagen‐coated glass coverslips in basal compartment. AF488‐labelled Bt‐BEVs (1 × 10^11^/mL) or PBS were added to the co‐culture system for 24 h (37°C, 5% CO_2_). (a) Representative UV confocal microscopy image of hCMEC/D3 cells following 24 h Bt‐BEV (green) incubation in gut‐BBB two‐cell culture system. Cells co‐stained with anti‐LAMP1 (red) and nuclear stain Hoechst 33342 (blue). Quantitative analysis of Bt‐BEV co‐localisation with intracellular lysosomes were calculated from 20 field of view (FOV) images from five biological replicates. (b) TEER measurements for Caco‐2 cell monolayer taken during 24 h following Bt‐BEV (*n* = 6) or PBS (*n* = 3) addition. Statistical significance calculated using the Mann–Whitney test, a value *p* < 0.05 was considered statistically significant (GraphPad Prism 5.04); *p < 0.05, **p < 0.01, ***p < 0.001. Scale bar = 25 μm.

To model gut epithelial and BBB endothelial cell monolayers, Caco‐2 and hCMEC/D3 cells were cultured together in a dual culture model (Figure 2). This consisted of ThinCert inserts with a polyethylene terephthalate (PET) membrane assembled with corresponding hCMEC/D3 monolayers cultured on glass coverslips (Figures 2b and 3) in the base of a 24‐well plate. Within 60 min of Bt‐BEV addition to the apical (luminal) compartment containing Caco‐2 cells, a transient increase in permeability, as demonstrated by reduced TEER measurements compared to PBS treated cells, was observed. After 24 h, this remained significantly higher from that of control cultures with a trend towards returning to baseline values (Figure 3b).

### 3.4 Bt‐BEVs do not elicit an inflammatory response in non‐differentiated neuronal cells in monocultures

**FIGURE 4:**
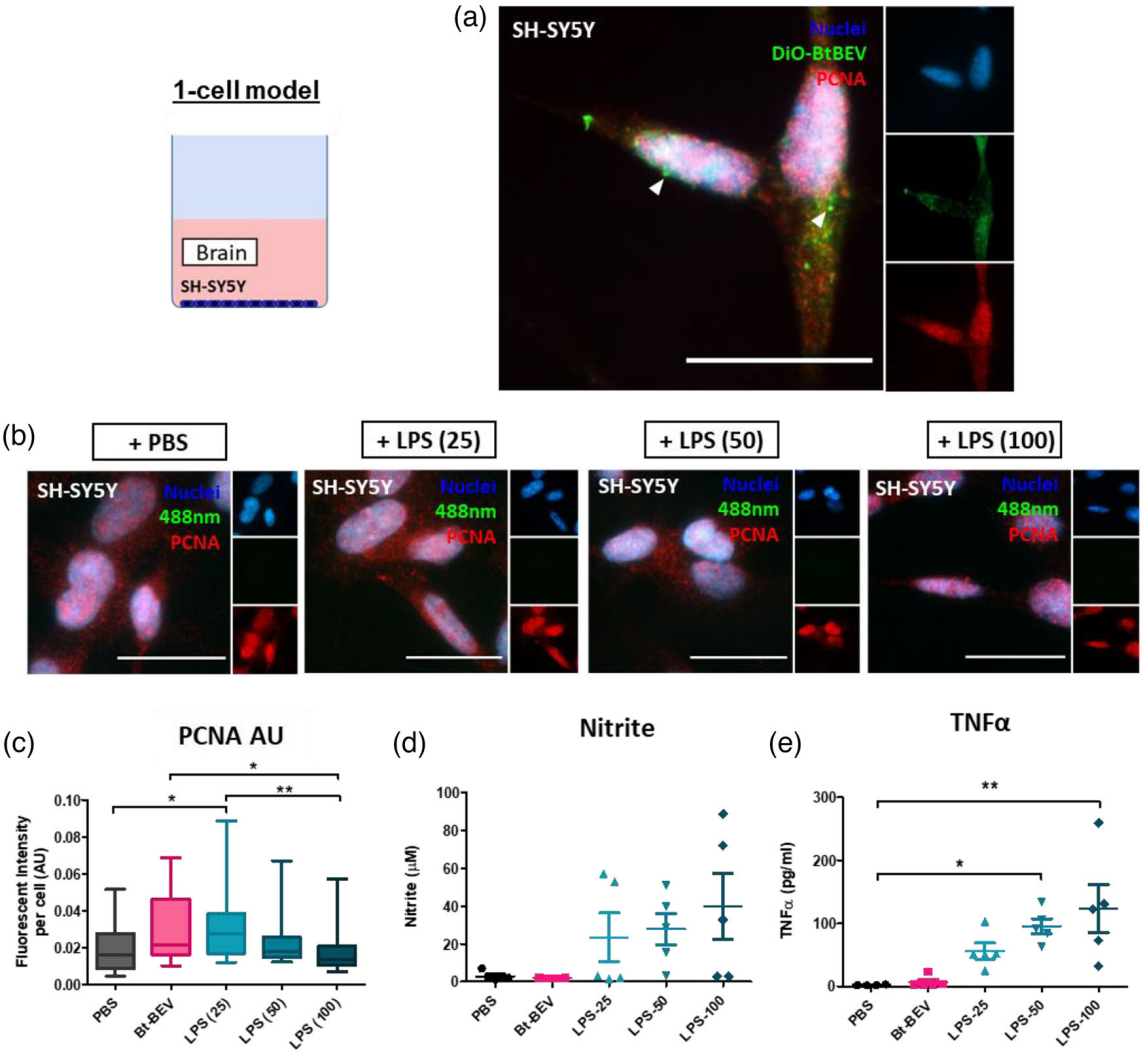
Bt‐BEVs do not appear to elicit an inflammatory response in non‐differentiated neuronal cells. Non‐differentiated SH‐SY5Y cell cultures were incubated with either PBS, DiO‐labelled Bt‐BEVs (1 × 10^11^/mL) or LPS (25, 50, 100 ng/mL) for 24 h (37°C, 5% CO_2_). Representative photomicrographs of (a) DiO‐BtBEV treated (green) and (b) PBS or LPS treated SH‐SY5Y cells fixed and co‐stained with anti‐PCNA (red) and Hoechst 33342 nuclear stain. White arrowheads indicate uptake of DiO‐labelled Bt‐BEVs. (c) Quantification of normalised mean fluorescent intensity of anti‐PCNA staining in SH‐SY5Y cells incubated with PBS, Bt‐BEVs and LPS. A minimum of 20 field of view (FOV) images from each group were used to calculate mean fluorescent intensity. The box plots represents first quartile, median and third quartile, with whiskers representing minimum and maximum. (d) Nitrite and (e) TNF‐α concentrations measured in SH‐SY5Y cell culture media following incubation with PBS (*n* = 4/5), Bt‐BEVs (*n* = 5), or LPS (*n* = 5). Data are represented as mean ± SEM. Statistical significance calculated using Kruskal–Wallis test (GraphPad Prism 5.04) with a value of *p* < 0.05 considered statistically significant; *p < 0.05, **p < 0.01, ***p < 0.001. Images taken on Zeiss AxioImager Widefield Fluorescent microscope (×40/objective). Scale bars = 25 μm.

To assess the response of immature neurons to Bt‐BEVs, SH‐SY5Y cells were incubated with either DiO‐labelled Bt‐BEVs, PBS, or a range of LPS concentrations (Figure 4). Within 24 h, Bt‐BEVs were acquired by SH‐SY5Y cells with a sub‐cellular localisation (Figure 4a). Quantification of the mean fluorescent intensity (AU) of anti‐PCNA staining normalised to nuclear counts revealed that Bt‐BEVs induced a small although non‐significant increase in fluorescent intensity of antibody staining compared to PBS (Figure 4c; 0.03 ± 0.003 AU compared to 0.02 ± 0.002 AU, respectively). By contrast, LPS treatment (at 25 ng/mL) significantly (*p* < 0.01) increased the fluorescent intensity of anti‐PCNA staining in SH‐SY5Y cells compared to PBS (Figure 4c; 0.03 ± 0.004 AU).

The levels of nitrite and TNF‐α in the cell culture supernatant following incubation with PBS or Bt‐BEVs were not significantly different (Figure 4d and e). This contrasted with LPS where a dose‐dependent increase in nitrite levels was observed although this did not reach significance (Figure 4d). Similar findings were observed for TNF‐α, with dose‐dependent increases in secretion of TNF‐α detected following LPS incubation, reaching significance (*p* < 0.05 and *p* < 0.01, respectively) at 50 and 100 ng/mL LPS (Figure 4e).

### 3.5 Bt‐BEVs cross BBB endothelial cells and are acquired by neuronal cells

**FIGURE 5:**
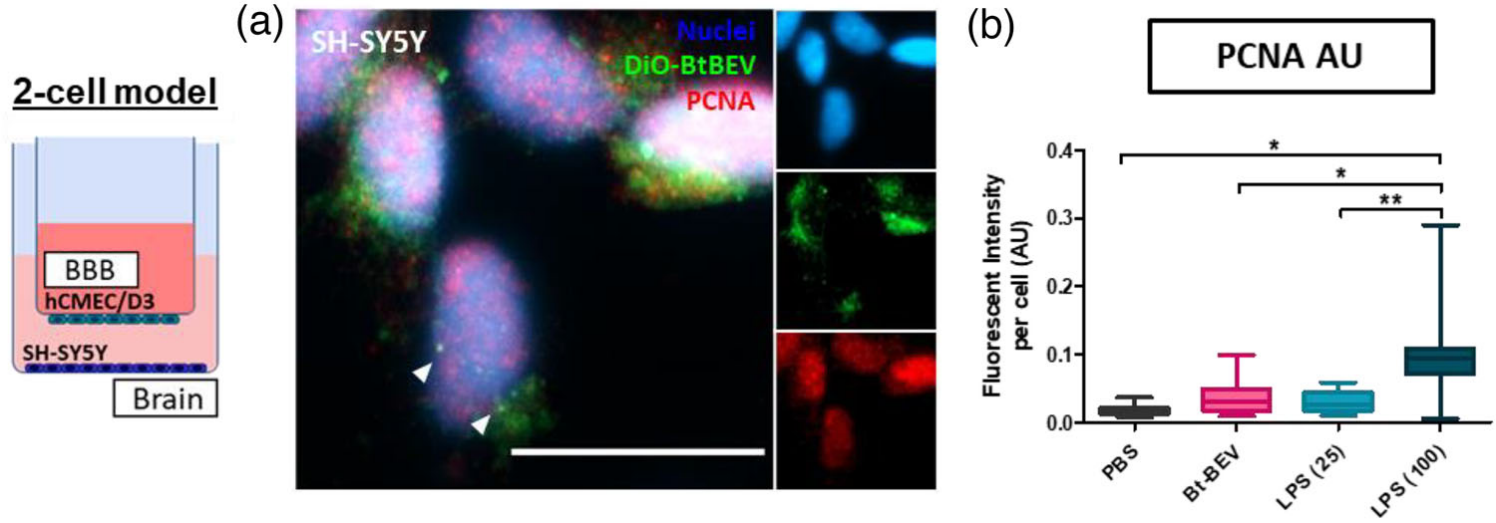
Bt‐BEVs cross the BBB endothelium and are acquired by neuronal cells. A two‐cell culture system was developed by culturing hCMEC/D3 cells to confluence on the underside of Matrigel‐coated ThinCert membranes and added to non‐differentiated SH‐SY5Y cells cultured on fibronectin‐coated glass coverslips in a 24 well plate. PBS, DiO‐labelled Bt‐BEVs (1 × 10^11^/mL) or LPS (25 and 100 ng/mL) were added to the apical compartment and the two‐cell culture system incubated for 24 h (37°C, 5% CO_2_). (a) Representative photomicrographs of SH‐SY5Y cells co‐stained with anti‐PCNA (red) following 24 h incubation with DiO‐labelled Bt‐BEVs (green). (b) Normalised mean fluorescent intensity (AU) of anti‐PCNA staining quantified from a minimum of 15 field of view (FOV) images from each group (*n* = 1). The box plots represents first quartile, median and third quartile, with whiskers representing minimum and maximum. Statistical significance calculated using Kruskal–Wallis test (GraphPad Prism 5.04) with a value of *p* < 0.05 considered statistically significant; *p < 0.05, **p < 0.01. Image taken on Zeiss AxioImager Widefield Fluorescent microscope (×40/objective). Scale bar = 25 μm.

The ability of Bt BEVs to transmigrate BBB endothelial cells and be acquired by neuronal cells was investigated using DiO‐labelled Bt‐BEVs added to hCMEC/D3 cells cultured in the apical compartment of ThinCert inserts with non‐differentiated SH‐SY5Y neuronal cells cultured on fibronectin‐coated glass coverslips in the corresponding basal compartment of a 24‐well plate (Figure 5). PBS and LPS were used as comparators. Bt‐BEVs were detected in SH‐SY5Y cells, indicative of the transmigration of Bt‐BEVs across the hCMEC/D3 cell monolayer (Figure 5a). By contrast, no fluorescent particles were detected in PBS or LPS treated two‐cell, hCMEC/D3 and SH‐SY5Y, culture systems (data not shown).

The impact of Bt BEVs on cycling neuronal cells was assessed by analysing the mean fluorescent intensity of anti‐PCNA staining normalised to nuclear counts. No significant difference was observed in staining following incubation with Bt‐BEVs when compared to PBS (Figure 5b; 0.04 ± 0.01 AU vs. 0.02 ± 0.001 AU, respectively). LPS at the lower concentration (25 ng/mL) also did not have a significant effect on PCNA fluorescent intensity, although a significant increase (*p* < 0.01) was observed following incubation with 100 ng/mL LPS (0.10 ± 0.01 AU). These results are consistent with the trafficking of BEVs to immature neuronal cells via transmigration of BBB enodthelial cells resulting in no discernable impact on cell cycling.

### 3.6 Bt‐BEVs activate microglia cells in monocultures

**FIGURE 6:**
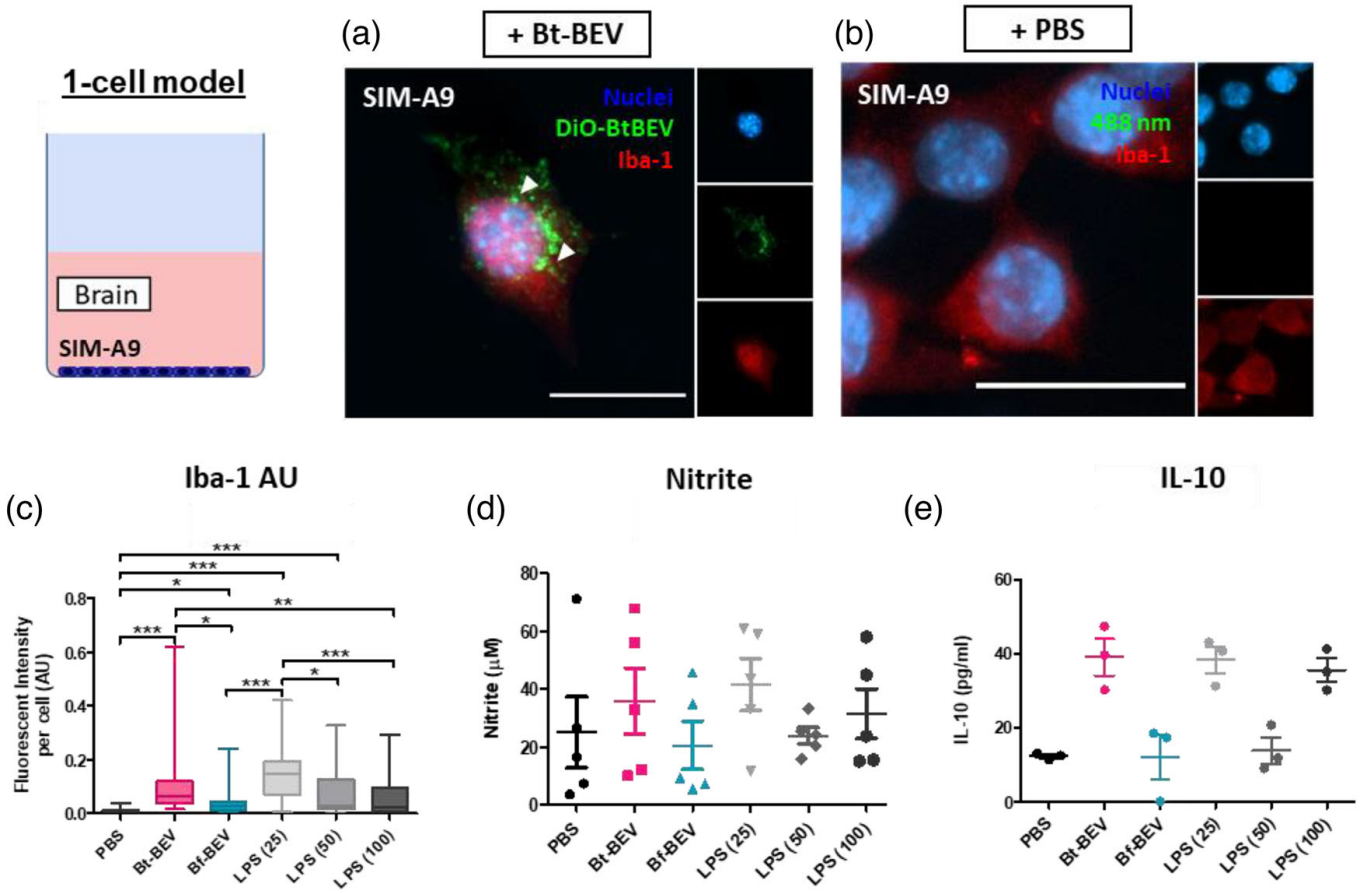
Bt‐BEVs are acquired by murine microglia and induce an activation and inflammatory response. SIM‐A9 cell cultures were incubated for 24 h (37°C, 5% CO_2_) with PBS, Bt‐BEVs (1 × 10^11^/mL), Bf‐BEVs (1 × 1011/mL) or LPS (25, 50, 100 ng/mL). Cells were then fixed and co‐stained with anti‐Iba‐1 and nuclear stain Hoechst 33342 (blue) prior to imaging by UV confocal microscopy. (a) DiO‐labelled Bt‐BEV acquisition (green, white arrowheads) in SIM‐A9 cells following 24 h incubation. Cells co‐stained with anti‐Iba‐1 (red). (b) PBS control treated SIM‐A9 cells co‐stained with anti‐Iba‐1 (red). (c) Normalised mean fluorescent intensity of Iba‐1+ staining in SIM‐A9 cells incubated with PBS (*n* = 1), Bt‐BEVs (*n* = 1), Bf‐BEVs (*n* = 1) or LPS (*n* = 1) for 24 h. Analysis conducted from minimum of 30 FOV images from one sample in each group. (d) Nitrite concentrations (μM) in SIM‐A9 culture media following 24 h incubation with PBS (*n* = 5), Bt‐BEVs (1 × 10^11^/mL; *n* = 5), Bf‐BEVs (1 × 10^11^/mL; *n* = 5) or LPS (25, 50, and 100 ng/mL; *n* = 5) were determined using Griess Reagent. (e) IL‐10 concentrations (pg/ml) in SIM‐A9 culture media following 24 h incubation with PBS (*n* = 3), Bt‐BEVs (1 × 10^11^/mL; *n* = 3), Bf‐BEVs (1 × 10^11^/mL; *n* = 3) or LPS (25, 50, and 100 ng/mL; *n* = 3) were determined using by ELISA. The box plots represent first quartile, median and third quartile, with whiskers representing minimum and maximum and the scatterplots represent mean ± SEM. Statistical significance was calculated using Kruskal–Wallis test followed by Dunn's multiple comparison test with a value of *p* < 0.05 considered statistically significant (GraphPad Prism 5.04); *p < 0.05, **p < 0.01, ***p < 0.001. Images taken on confocal microscope (63×/1.4 oil DIC objective). Scale bars = 25 μm.

To investigate uptake of BEVs by other CNS cell types, monocultures of the murine immortalised microglia cell lines SIM‐A9 and BV‐2, were used. DiO/DiD‐labelled Bt‐BEVs were acquired by SIM‐A9 (Figure 6) and BV‐2 cells (Figure S4) within 24 h of exposure, displaying a perinuclear localisation. In addition, similar patterns of BEV lysosomal localisation to those observed in hCMEC/D3 cells (Figure S2E) were observed in BV‐2 cells as evidenced by co‐staining with anti‐LAMP1 antibodies (Figure S4B). Staining with the F‐actin stain phalloidin‐488 revealed membrane ruffling characterised by retraction of long multidirectional cellular projections and “cupping” of the membrane (Figure S4C) consistent with cellular activation (Durant et al., 2020; Gao et al., 2009; López‐Carballo et al., 2002; Yoo et al., 2016). Activation of microglial cells was confirmed by expression of the activation‐associated antigen Iba‐1 cells following exposure to Bt‐BEVs, revealing a significant increase (*p* < 0.001) in the intensity of Iba‐1 staining per cell compared to PBS treated cells (Figure 6c; 0.11 ± 0.02 AU vs. 0.01 ± 0.001 AU). The activation response elicited by Bt‐BEV was more apparent than that observed with BEVs from *B. fragilis* (Bf) or LPS. Analysis of nitrite concentration revealed no obvious effect of Bt‐BEVs on oxidative stress (Figure 6d).

We have previously shown that Bt‐BEVs elicit production of the hallmark anti‐inflammatory cytokine IL‐10 by colonic and blood‐derived human dendritic cells (Durant et al., 2020). To assess whether Bt‐BEVs were able to promote a similar regulatory response in microglial cells, IL‐10 secretion were measured in supernatants from SIM‐A9 cells after co‐culture with Bt‐BEVs, PBS or LPS (Figure 6e). Bt‐BEVs induced an increase in IL‐10 secretion compared to PBS (39.1 ± 4.98 pg/mL compared to 12.4 ± 0.49 pg/mL) although it did not reach statistical significance. This immunoregulatory response in SIM‐A9 cells was not observed in cells incubated with Bf‐BEVs or with 50 ng/mL of LPS with levels of IL‐10 being comparable to that in PBS cultures (13.9 ± 3.52 pg/mL) and lower than that seen in cultures exposed to Bt‐BEVs.

### 3.7 Bt‐BEVs can transmigrate across intestinal epithelial and BBB endothelial cell barriers and be acquired by neuronal cells

Prior to establishing the parameters of a three‐cell‐culture system, the optimal culture conditions to obtain a confluent monolayer of hCMEC/D3 cells were evaluated using TEER measurements. Matrigel was superior to collagen or no supporting matrix in producing the highest TEER values (∼300 Ωcm^2^) (Fig S5A). Matrigel also reduced the transmigration of BEVs through ThinCert cultures of hCMEC/D3 cells when compared to collagen coating (data not shown), consistent with Matrigel providing higher TEER values and reduced permeability. Matrigel was therefore used to culture hCMEC/D3 cells in multi‐cell culture systems.

**FIGURE 7:**
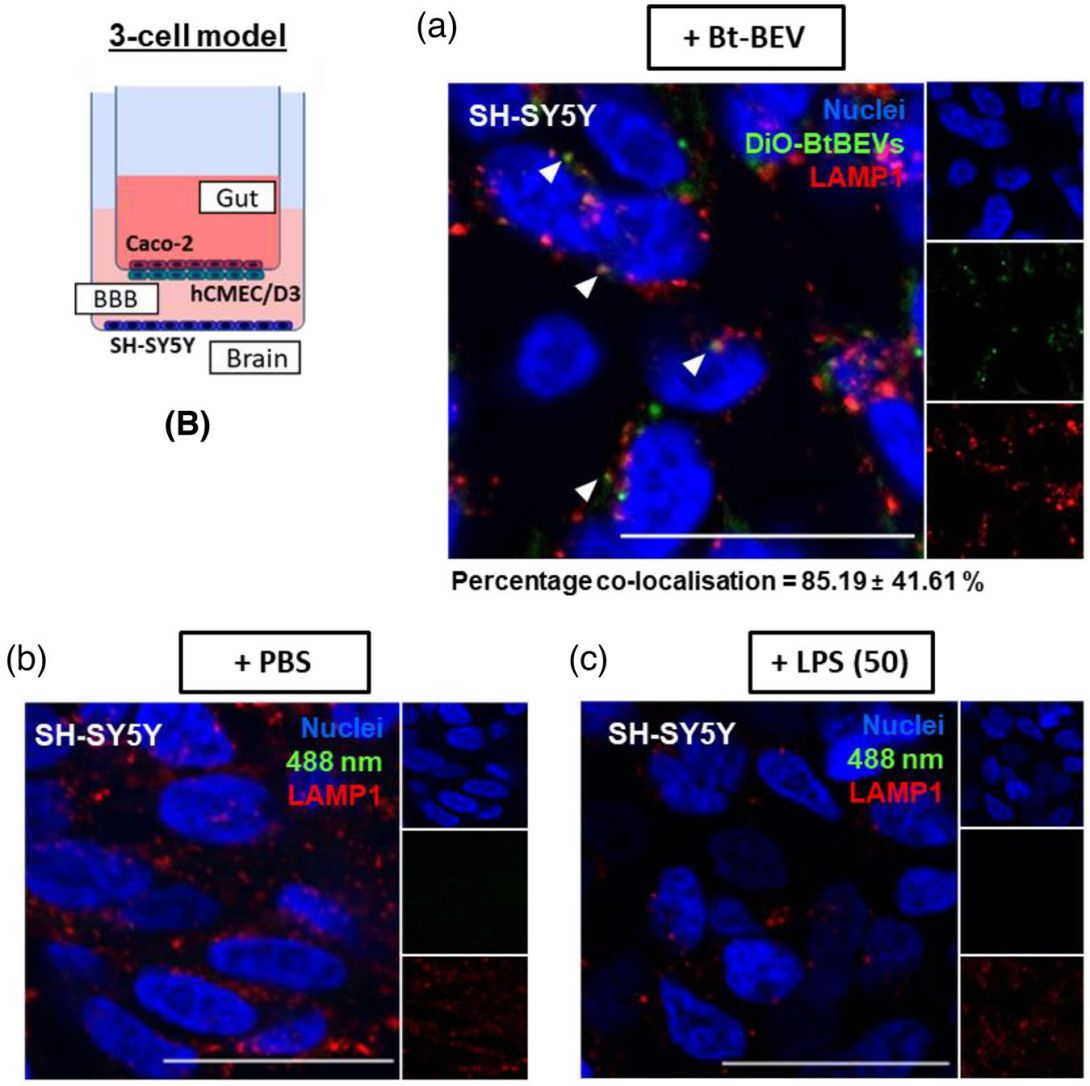
Bt‐BEVs cross the gut epithelial and BBB endothelial cell barriers and are acquired by non‐differentiated neurons. A three‐cell culture system was set up with Caco‐2 and hCMEC/D3 cells modelling the gut‐brain barriers cultured on ThinCert inserts (PET membrane, 0.4 μm), and non‐differentiated SH‐SY5Y cells cultured on fibronectin coated glass‐coverslips in the basal compartment. DiO‐labelled Bt‐BEVs (1 × 10^11^/mL), PBS or LPS (50 ng/mL) were added to the apical compartment of the three‐cell culture system for 24 h (37°C, 5% CO_2_) prior to antibody staining and UV confocal microscopy. SH‐SY5Y cells were fixed and co‐stained with anti‐LAMP1 (lysosomes, red) and nuclear stain Hoechst 33342 (blue). (a) Co‐localisation of Bt‐BEVs (green) with intracellular lysosomes (red) in SH‐SY5Y cells indicated by white arrowheads. (b) PBS and (c) LPS controls showing intracellular lysosomes (red) and absence of fluorescent signal in 488 nm channel. Images taken on confocal microscope (63×/1.4 oil DIC objective). Scale bars = 25 μm.

The three‐cell culture system utilised Caco‐2 and hCMEC/D3 cells cultured on the apical and basal side of ThinCert PET 0.4 μm membranes and non‐differentiated SH‐SY5Y cells cultured on glass coverslips in the base of a 24 well plate. This three‐cell system was used to model Bt‐BEV transmigration from the gut through the BBB and into the CNS (Figure 2c). Using TEER as an indicator of confluency and permeability of Caco‐2 and hCMEC/D3 cells cultured on the ThinCert PET membrane measurements were taken daily for 21 days following Caco‐2 and hCMEC/D3 seeding on the basal side of PET membrane at day 18 (Figure S5B). Thincert inserts were subsequently placed in corresponding wells containing non‐differentiated SH‐SY5Y cells. Bt‐BEVs added to the apical surface of Caco‐2 cells were acquired by SH‐SY5Y cells and sequestered to endo‐lysosomal pathways, identified by co‐staining with anti‐LAMP1. The vast majority (85.19 ± 41.61%) of internalised Bt‐BEVs co‐localised with lysosomes (Figure 7a). No equivalent fluorescence signal was observed in PBS or LPS treated cultures (Figure 7b and c).

**FIGURE 8:**
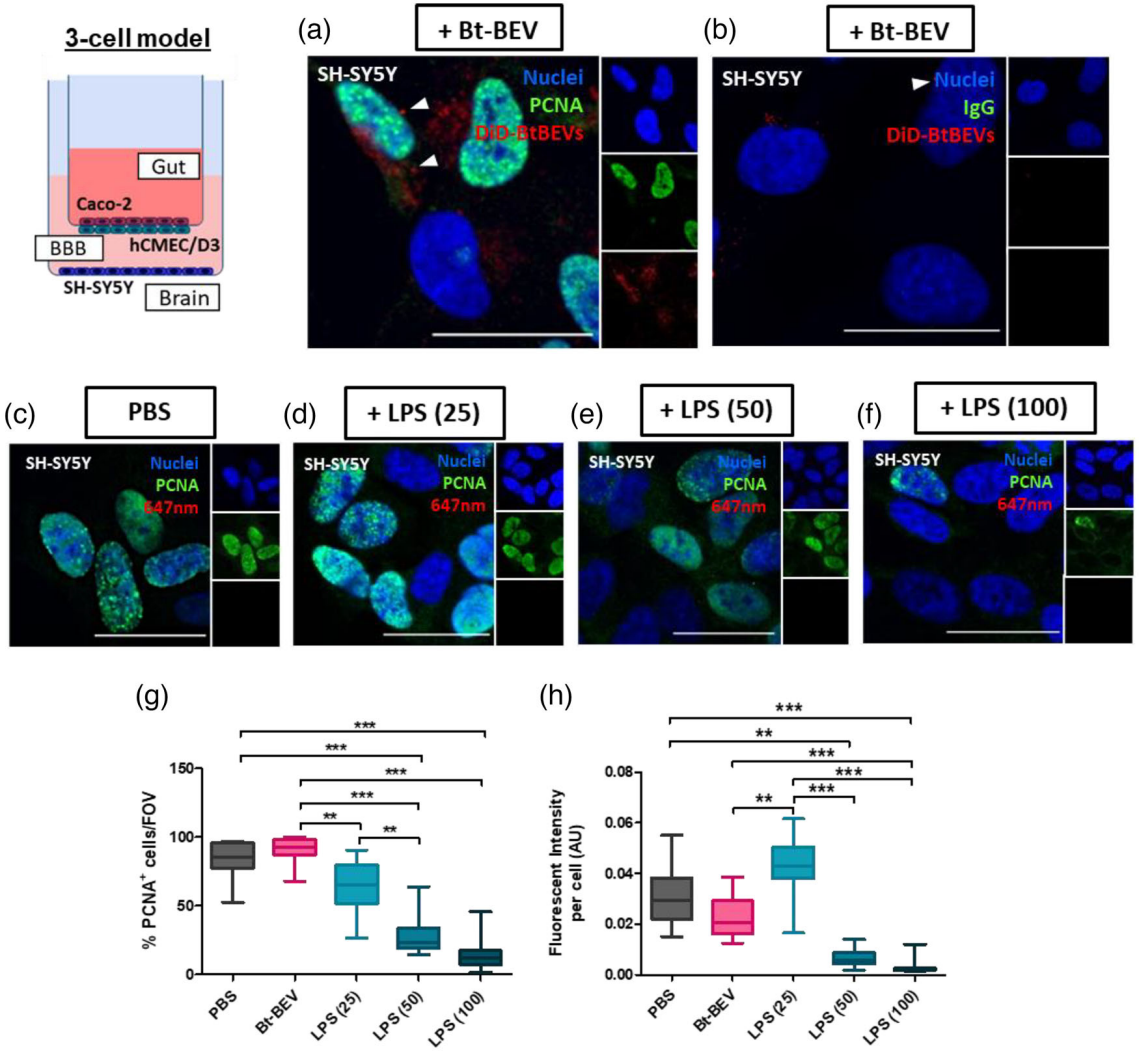
LPS, but not Bt‐BEVs, alters cell cycling activity of non‐differentiated neuronal cells. A three‐cell culture system containing Caco‐2 and hCMEC/D3 cells modelling the gut‐brain barriers cultured on ThinCert inserts (PET membrane, 0.4 μm) and non‐differentiated SH‐SY5Y cells cultured on fibronectin coated glass‐coverslips in the basal compartment. DiD‐labelled Bt‐BEVs (1 × 10^11^/mL), PBS or LPS (25, 50, 100 ng/mL) were added to the apical compartment of the three‐cell culture system for 24 h (37°C, 5% CO_2_). SH‐SY5Y cells were then fixed and co‐stained with anti‐PCNA (green), a marker for cell proliferation and nuclear stain Hoechst 33342 (blue) and imaged by UV confocal microscopy. Representative images of SH‐SY5Y cells from (a) PBS, (b) 25 ng/mL LPS, (c) 50 ng/mL LPS, (d) 100 ng/mL LPS and (e) Bt‐BEV treated three‐cell culture system. No fluorescent 647 nm signal was observed in PBS or LPS treated cells. (f) IgG was used as an isotype control. (g) Percentage of total PCNA^+^ SH‐SY5Y cells from PBS, Bt‐BEV and LPS treated three‐cell culture systems. Cell counts conducted from minimum of 20 field of view (FOV) images and represented as percentage of total cells. (h) Mean fluorescent intensities normalised to nuclear counts of anti‐PCNA staining in SH‐SY5Y cells from the three‐cell culture system treated with PBS, Bt‐BEVs or LPS. Analysis conducted from minimum of 20 FOV images. The box plots represents first quartile, median and third quartile, with whiskers representing minimum and maximum. Statistical significance determined using Kruskal–Wallis multiple comparisons, a value p < 0.05 was considered statistically significant (GraphPad Prism 5.04); *p < 0.05, **p < 0.01, ****p < 0.0001. Images taken on confocal microscope (63x/1.4 oil DIC objective). Scale bars = 25 μm.

**FIGURE 9:**
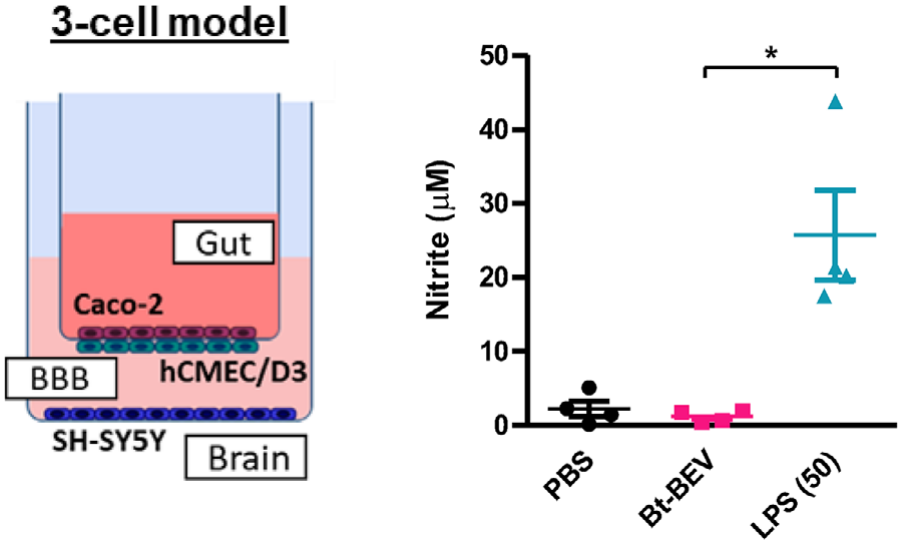
LPS induces nitrite production in a three‐cell culture system modelling the gut‐brain axis. A three‐cell culture system was set up with Caco‐2 and hCMEC/D3 cells modelling the gut‐brain barriers cultured on ThinCert inserts (PET membrane, 0.4 μm) and undifferentiated SH‐SY5Y cells cultured on fibronectin coated glass‐coverslips in the basal compartment. DiD‐labelled Bt‐BEVs (1 × 10^11^/mL), PBS or LPS (50 ng/mL) were added to the apical compartment of the three‐cell culture system for 24 h (37°C, 5% CO_2_). Nitrite (μM) was measured in media from the basal compartment following incubation with PBS (*n* = 4), Bt‐BEV (*n* = 4) or LPS (*n* = 4) using Griess Test. The scatter dot‐plots represent individual values and mean ± SEM. Statistical significance determined using Kruskal–Wallis multiple comparisons, a value *p* < 0.05 was considered statistically significant (GraphPad Prism 5.04); *p < 0.05, ns. not significant.

To determine the effects of Bt‐BEVs on cycling cells SH‐SY5Y cells from the three‐cell culture system were analysed for PCNA expression (Figure 8a–f). No significant difference in PCNA^+^ cells were observed between PBS and Bt‐BEV treated cultures, consistent with Bt‐BEVs transmigration across the epithelial and endothelial cell barriers having no impact on SH‐SY5Y cell cycle and proliferation (Figure 8g). Interestingly, fluorescent intensities of PCNA^+^ staining showed no significant alterations per cell in PBS and Bt‐BEV treated cells (Figure 8h; 0.03 ± 0.002 and 0.02 ± 0.001, respectively). By contrast, LPS treatment (25, 50, and 100 ng/mL) significantly (*p* < 0.001) reduced the proportion of PCNA^+^ cells (63.19 ± 3.84%, 27.79 ± 2.69%, and 14.98 ± 2.40%, respectively) compared to Bt‐BEV treatment (Figure 8g; 91.81 ± 1.71%) in parallel with significant (*p* < 0.001) reductions in fluorescent intensity per cell at 100 ng/mL concentration (0.003 ± 0.001 AU) compared to Bt‐BEV treated cultures (0.02 ± 0.01 AU). A similar pattern was seen in nitrite production (Figure 9) with no significant difference in production by SH‐SY5Y cells in Bt‐BEV treated cultures (1.20 ± 0.35 μM), whereas LPS (50 ng/mL) treatment resulted in a significant (*p* < 0.05) increase in nitrite production (25.72 ± 4.96 μM).

To determine if Bt‐BEVs impact on the differentiation of SH‐SY5Y cells, they were stained post‐BEV exposure with antibodies specific for Neuro D1 (ND1), a transcription factor upregulated during neurogenesis and differentiation of SH‐SY5Y cells (López‐Carballo et al., 2002) that is important in the maturation and survival of neurons in the hippocampus and olfactory bulb (Gao et al., 2009) (Figure S6A‐C). Quantification of fluorescent intensity of ND1 staining (Figure S6D) revealed no significant differences in mean fluroscent intensity per cell between PBS and Bt‐BEV treated cultures. However, LPS caused a small but non‐significant increase in ND1 expression (0.01 ± 0.002 AU). Overall, our findings indicate that Bt‐BEVs, in contrast to LPS, do not elicit anti‐proliferative or inflammatory responses in non‐differentiated SH‐SY5Y cells following their transmigration across gut epithlial and BBB endothelial cell barriers.

## 4 DISCUSSION

Here we demonstrate the potential for Bt BEVs to access neuronal cells *in vivo* in the brains of mice and *in vitro* using a multi‐cell culture model of the gut‐brain axis. Following oral or intravenous administration fluorescently labelled BEVs were detected in several organs including the brain (Jones et al., 2020). Although the vast majority of BEVs likely remain in the circulatory system (Jones et al., 2021), a proportion will interact with and transmigrate intestinal epithelial and vascular endothelial cell barriers and the BBB endothelial barrier to reach underlying tissues, or are acquired intracellularly via endocytic pathways (Jang et al., 2015; Jones et al., 2020; Yoo et al., 2016). To investigate further how BEVs interact with these cell barriers we developed a simplified *in vitro* multi‐cell culture model comprising major barrier cell types of the gut‐brain axis. Exposure of cell monocultures to Bt‐BEVs resulted in their uptake and intracellular trafficking via endo‐lysosomal pathways in both BBB endothelial and neuronal cells with induction of downstream pro‐ or anti‐inflammatory responses depending on the cell type. A three‐cell culture system incorporating Caco‐2 and hCMEC/D3 cells to model the gut epithelium and BBB endothelium, respectively, and non‐differentiated SH‐SY5Y neuronal cells demonstrated that Bt‐BEVs can via transmigration of epithelial and endothelial cell monolayers be acquired by, and localise to intracellular lysosomes of neuronal cells. Comparing downstream effects of Bt‐BEV acquisition in SH‐SY5Y cells with endotoxin (*E. coli*‐derived LPS) indicated that Bt‐BEVs do not induce significant changes in proliferative activity, differentiation, or elicit pro‐inflammatory responses in non‐differentiated neuronal cells. These findings have ramifications for the potential use of BEVs as drug delivery vehicles and delivering therapeutics to the CNS.

It should be noted that although culturing epithelial/endothelial cells as a monoculture on a solid support is widely used to locate, identify and image cells, it is limited in replicating *in vivo* physiology and they exclude the contribution of other cells that are part of and contribute to gut epithelial (e.g., immune, and enteric nervous system cells) and CNS endothelial (e.g., astrocytes and pericytes) barrier integrity and permeability (Sivandzade & Cucullo, 2018). The ThinCert system allows for the apical or basal culture of cells on a microporous semi‐permeable membrane, providing separation between the compartments. Here we used this system to co‐culture cells and mimic the luminal, vascular, and parenchymal compartments. The detection of Bt‐BEVs in the basal compartment of the two‐cell (gut‐BBB) model and their subsequent internalisation by hCMEC/D3 cells indicates the ability of Bt‐BEVs to transmigrate a barrier comprising a monolayer of epithelial cells. Interestingly, lysosomal co‐localisation was higher in this co‐culture model compared to monocultures. Despite the utility and usefulness of Caco‐2 cells to investigate various aspects of intestinal epithelial cell physiology, they cannot faithfully replicate the inherent cellular complexity and the different epithelial cell lineages present within the intestinal epithelium *in vivo*.

The BBB is a highly regulated barrier controlling the exchange between the blood and brain compartments (Abbott et al., 2010) and the hCMEC/D3 cells have been widely used to model the human BBB endothelial cells (Weksler et al., 2013). Tight junctions in BBB endothelial cells, comprised of proteins including claudin‐5 and ZO‐1, are responsible for the selective permeability and high TEER values. However, claudin‐5 and ZO‐1 are present at a lower level in hCMEC/D3 cells and may have a different cellular distribution (Urich et al., 2012; Weksler et al., 2005). Also, claudin‐5 helps prevent small molecules (< 800 D) crossing the BBB (Nitta et al., 2003) and aids regulation of paracellular diffusion (Ohtsuki et al., 2007). Moreover, the ratio of claudin‐5 to claudin‐12 is important for the formation of tight junctions and is altered in hCMEC/D3 cells (Daneman et al., 2010; Ohtsuki et al., 2007; Urich et al., 2012). Of relevance to our study, hCMEC/D3 cells display a 5‐fold higher level of tight junction proteins on transwell filters compared to plastic coverslips (Weksler et al., 2013). Recent attempts have been made to develop the barrier properties of hCMEC/D3 cells by co‐culturing with other cells of the neurovascular unit (Hatherell et al., 2011) but the improvements are minor and additional development is required. Here, after initially establishing that the use of Matrigel as the supporting basement matrix for hCMEC/D3 cells significantly increased their TEER values, these cultures were incorporated into co‐cultures with SH‐SY5Y or BV‐2 cells to explore BBB transmigration of Bt‐BEVs and their interaction with neuronal cells (Figure 2b). Our results show that Bt‐BEVs can cross the BBB endothelium and be acquired by neuronal and microglial cells. Similar findings were observed *in vivo* following intravenous administration of *A. actinomycetemcomitans* BEVs with their accumulation in brain microglia cells (Ha et al., 2020).

Recent studies have shown commensal BEVs elicit immunomodulatory responses in monocytes/macrophages *in vitro* (Hu et al., 2020) and *ex vivo* (Durant et al., 2020) the signature of which is IL‐10 production. Using BV‐2 microglia cells, we have shown that they acquire Bt‐BEVs resulting in cellular activation and production of IL‐10. Interestingly, BEVs produced by a related *Bacteroides* species, *B. fragilis*, did not elicit an IL‐10 response from the neuronal cells suggesting the immunoregulatory effects of BEVs may be species specific. By contrast, LPS, the main structural component of the outer membrane of gram‐negative bacteria and in particular *E. coli*, (Raetz et al., 2002) elicits a robust and dose‐dependent pro‐inflammatory response and increase in nitrite and TNF‐α in non‐differentiated SH‐SY5Y cells, which was not seen in Bt‐BEV neuronal cell cultures. Similar contrasting findings of Bt BEV and LPS exposure are seen in three‐cell culture systems with LPS. Differences in response to Bt‐BEVs versus LPS most likely relate to differences in the liposaccharides produced by different gram‐negative bacteria. *Bacteroides thetaiotaomicron* VPI 5482 produces lipooligosaccharides (LOS) that is structurally distinct from classical LPS (Badi et al., 2020; Jacobson et al., 2018; Raetz et al., 2002). Bt‐BEVs and LPS do, however, share similarities in activation of toll‐like receptor 4 (TLR‐4) (Bäckhed et al., 2003; Gul et al., 2022) although Bt BEVs mainly activate innate immune cells via TLR‐2 mediated activation (Fonseca et al., 2022).

BEV preparations are heterogenous, containing different populations of similar sized microvesicles including predominantly outer membrane vesicles (OMVs) produced by viable bacteria as well as outer‐inner membrane vesicles and explosive outer membrane vesicles produced as a result of cell lysis (Juodeikis & Carding, 2022). It is not possible therefore to determine if there is any selectivity amongst BEVs in their ability to transmigrate epithelial/endothelial barriers and impact on neuronal cell function. Harvesting BEVs during the late log to early stationary phase of Bt growth cycle should ensure that the BEVs used in this study consist primarily of OMVs.

Finally, the use of fluorescent dyes to visualise/quantify BEVs in cells/tissues can be problematic. Potential confounding issues are BEV aggregation (Gangadaran et al., 2018), formation of micelles in the solution due to the highly lipophilic nature of the dyes (Morales‐Kastresana et al., 2017) and leaching of fluorescent membrane dye to other intracellular membranes (Mulcahy et al., 2014) can make it difficult to distinguish between lipophilic‐labelled vesicles or contaminated artifacts results in differences in uptake (Takov et al., 2017).

## 5 CONCLUSION

Here we demonstrate the transport of BEVs derived from the human commensal Bt, across the gut‐epithelial barrier and their *in vivo* distribution in peripheral tissues. Using *in vitro* multi‐cell model of the gut‐brain axis, we further demonstrated the transmigration of Bt‐BEVs across gut‐epithelial and BBB endothelial barrier and their internalisation by neural cells to affect predominantly anti‐inflammatory, regulatory responses.

## AUTHOR CONTRIBUTIONS

All authors conceived and designed the experiments. Amisha A. Modasia, L. Ashley Blackshaw, Emily J. Jones and Simon R. Carding wrote the manuscript and Simon R. Carding and L. Ashley Blackshaw supervised the research. Amisha A. Modasia and Emily J. Jones executed the experimental work. All authors carried out data interpretation. Amisha A. Modasia and Emily J. Jones carried out statistical analysis. All authors revised, read, and approved the final manuscript.

## CONFLICT OF INTEREST STATEMENT

The authors declare that the research was conducted in the absence of any commercial or financial relationships that could be construed as a potential conflict of interest.

## Supporting information

Supplementary Figure 1. Bt‐BEV labelling workflow.
Supplementary Figure 2. Bt‐BEVs are acquired by BBB endothelial cells and localise to lysosomes.
Supplementary Figure 3. Intracellular Bt‐BEVs are detected in various focal planes in cells in BBB endothelial cells.
Supplementary Figure 5. Optimisation of the multi‐cell culture system.
Supplementary Figure 6. Bt‐BEVs do not impact differentiation of neuronal cells.
